# Case Report: Computed tomography-guided diagnosis and surgical removal of intraorbital salivary choristoma confirmed with cytology and histopathology

**DOI:** 10.3389/fvets.2025.1636470

**Published:** 2025-07-23

**Authors:** Catharine Morgan, Janny V. Evenhuis, Sarah Zurbuchen, Natalia Vapniarsky, Kelsey Brust

**Affiliations:** ^1^Veterinary Medical Teaching Hospital, University of California, Davis, Davis, CA, United States; ^2^Department of Pathology, Microbiology, and Immunology, School of Veterinary Medicine, University of California, Davis, Davis, CA, United States; ^3^Department of Surgical and Radiological Sciences, School of Veterinary Medicine, University of California, Davis, Davis, CA, United States

**Keywords:** salivary, choristoma, heterotopic, computed tomography, ectopic salivary gland tissue

## Abstract

A 10-year-old male castrated Golden Retriever was presented for evaluation of a 2-year history of a slowly progressing intraorbital mass. Computed tomography showed a large, ovoid, fluid-filled structure that was not clearly associated with the salivary or lacrimal glands. Cytology was most consistent with a mildly inflamed salivary lesion. Surgical removal with biopsy showed that the mass was a cyst-like structure with multifocal serous salivary gland tissue. Histopathologic diagnosis, combined with three-dimensional imaging confirmation of an abnormal anatomic location, is most consistent with salivary choristoma.

## Introduction

Salivary tissues are typically located within the major salivary glands (composed of bilateral parotid, mandibular, zygomatic, and sublingual glands) and minor salivary glands, which are a diffuse network of salivary tissue located in the tongue and mouth (1). Rarely, normal salivary tissue can be found outside of these normal locations. This is referred to as salivary choristoma, heterotopic salivary gland tissue (HSGT), or ectopic salivary tissue (2). These terms are commonly used interchangeably and describe the same anomaly of histologically normal salivary tissue found in an abnormal anatomic location and not associated with major salivary glands.

HSGT has been documented in various areas of the human body, including the middle ear, thyroid, gingiva, and cervical lymph nodes (3). The underlying embryologic mechanism remains speculative, but it is known that salivary tissue forms from invaginated endoderm, with further development of distal structures into secretory cells (2, 4). The histologic characteristics of salivary glands vary based on location (5). However, general characteristics include columnar epithelium and secretory acinar cells.

This report describes diagnostic imaging, treatment, and histopathological features of intraorbital salivary choristoma in a canine patient, which collectively have not been previously documented. This case expands current knowledge of heterotopic salivary gland tissue in veterinary medicine and underscores the need for further research into the prevalence, embryologic origins, and clinical implications of salivary choristomas.

## Case description

A 10-year-old male castrated Golden Retriever was presented to the University of California, Davis Veterinary Medical Teaching Hospital (UCD VMTH) Ophthalmology Department for evaluation of an intraorbital mass that had appeared 2 years prior. One month before presentation, he had been evaluated at an ophthalmology specialty practice, where they recommended further workup with computed tomography (CT) at a referral hospital. The primary veterinarian prescribed one drop of Neopolydex (Neomycin Sulfate 3.5 mg/mL, Polymyxin B Sulfate 10,000 units/mL, and Dexamethasone 0.1%) in the affected left eye twice per day. This reduced conjunctival hyperemia but did not reduce the size of the mass. The patient had a history of a complicated crown fracture of the left maxillary fourth premolar tooth (208), which was treated with extraction 2 years prior to presentation. He also had a history of cruciate disease, which was treated with tibial plateau leveling osteotomy (TPLO). On presentation to UCD VMTH, the patient weighed 46 kg and had normal vital parameters. The patient had a 4-cm flocculent orbital mass that anteriorly displaced the ventral portion of the left orbital rim and posteriorly displaced the left globe. The left eye also displayed mild serous discharge, mild enophthalmos, mild hyperemia of the third eyelid, low intraocular pressure (7 mmHg), and a negative Jones 1 test (lacrimal duct patency test). Ocular testing of the right eye was unremarkable. A complete blood count (CBC), chemistry, urinalysis, and thoracic radiographs were performed, none of which showed significant evidence of underlying disease or metastasis.

**Figure 1:**
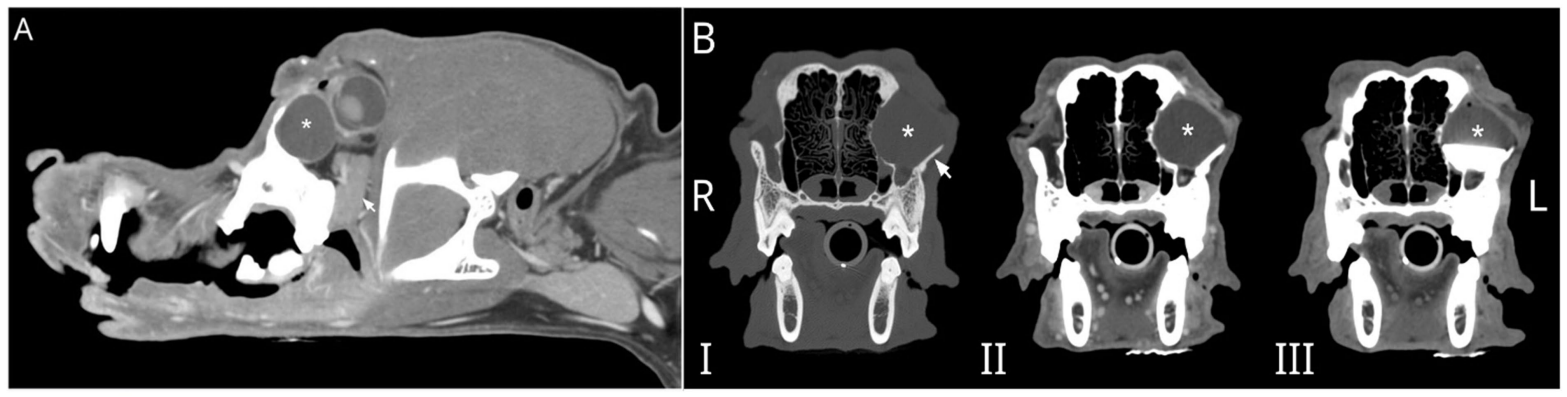
**(A)** Parasagittal reconstruction of the skull of a 10-year-old male castrated Golden Retriever post-IV contrast with a large, ovoid, fluid-filled structure (*) caudally displacing the globe. The zygomatic salivary gland (arrow) is ventral to the fluid-filled structure. **(B)** Multiphase transverse CT images show that this large, ovoid, fluid-filled structure (*) causes architectural changes to the left zygomatic arch (arrow). Dacrorhinocytography did not document contrast drainage into the intraorbital structure and did not drain into the adjacent canal (not shown) when contrast was directly injected into the structure in C. I- precontrast, II- post IV contrast, and III- post dacrorhinocytogram and direct injection into an ovoid fluid-filled structure.

The dog underwent general anesthesia for conventional computed tomography (CT) (LightSpeed16, General Electric Co., Milwaukee, WI) of the skull with the patient positioned in sternal recumbency. Scanning parameters were 150 mAs and 120 kV, collimation of 10 × 0.62 mm, pitch of 0.9375, and rotation time of 1 s. Images were obtained at a slice thickness of 0.62 mm. Initial CT scanning was followed by dacrocystography with approximately 2 mL of iopamidol (Isovue 370, Bracco Diagnostics, Monroe Township, NJ), followed by systemic contrast administration (800 mg/kg, IV). Finally, iopamidol was directly injected into the lesion (Figure 1). The imaging revealed a large, ovoid, fluid-filled structure measuring an average of 17 Hounsfield units (HUs), causing architectural changes to the zygomatic arch and caudally displacing the globe. The structure was not associated with the previously vacated alveolus of the left maxillary fourth premolar tooth. There was associated retropharyngeal lymph node asymmetry, with the left measuring 8.2 mm compared to 6.7 mm on the right. Dacrorhinocytography was performed via the inferior lateral punctum, which drained to the nasal cavity but did not drain into the intraorbital structure. Thick, stringy fluid was aspirated from the lesion, and cytology performed by clinical pathologists was most consistent with saliva with mild inflammation. The patient was referred to UCD VMTH Dentistry and Oral Surgery Service (DOSS) for surgical removal.

**Figure 2:**
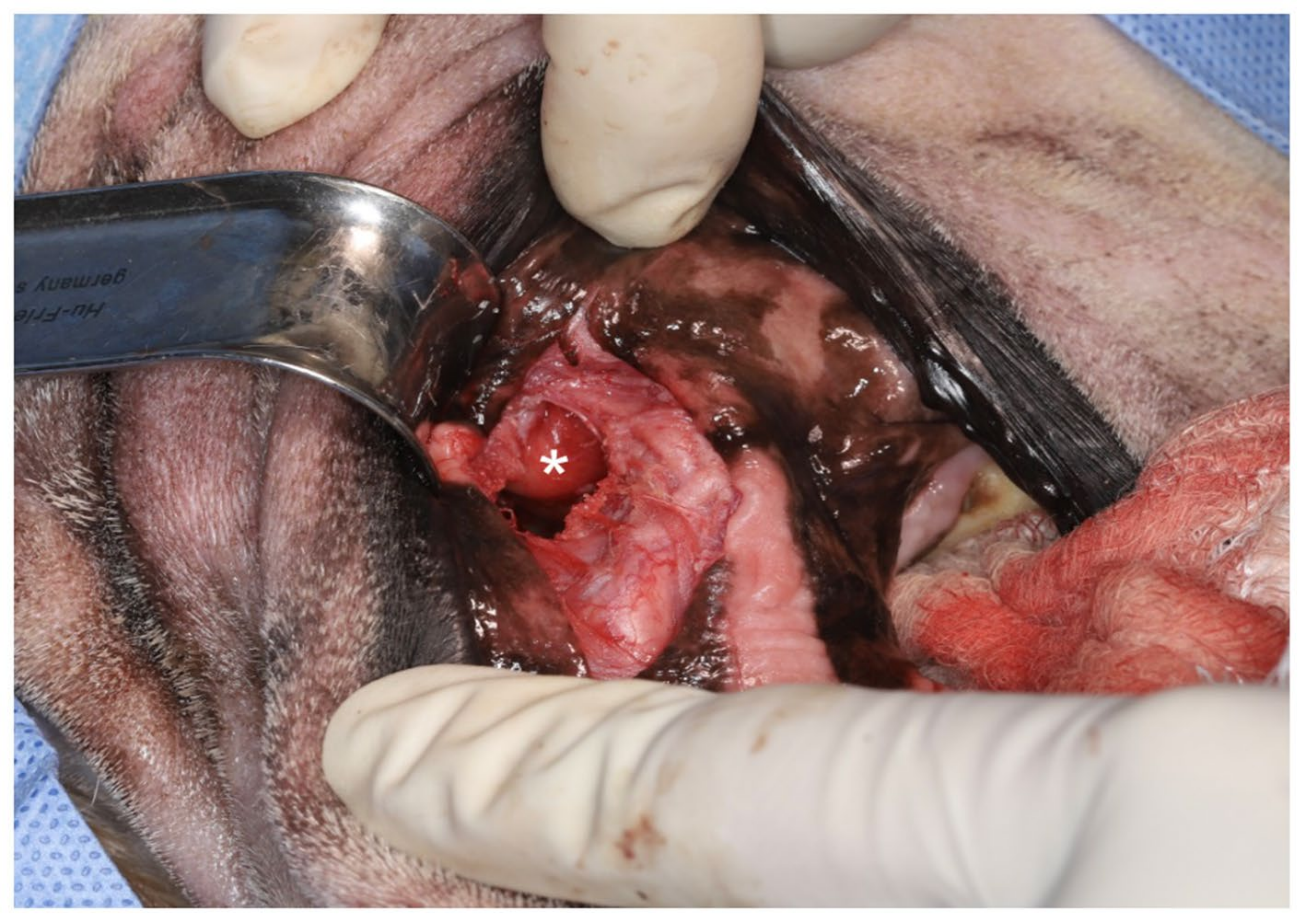
Intraoral surgical approach highlighting the opened, drained salivary mass, which had been debrided and flushed (*).

On presentation for surgery, CBC and chemistry were similar to the previous visit. For surgical removal, the patient was placed in right lateral recumbency and sterilely draped. Utilizing an intra-oral approach, a #15 blade was used to make an incision on the alveolar mucosa dorsal to the region of the left maxillary fourth premolar tooth and left maxillary first molar tooth (208–209). Using a combination of blunt and sharp dissection, the underlying expanded bone was exposed. A piezotome unit (Piezotome® Cube, Acteon, Mérignac, France) was used to create an approximately 1.5 cm x 1 cm window into the side of the mass (Figure 2). Copious yellow, clear, viscous fluid was suctioned from the surgical site. A Miller’s curette (Hu-Friedy, Chicago, IL) and a 24G periosteal elevator (Hu-Friedy, Chicago, IL) were used to gradually remove the cyst lining in pieces until no visible cyst lining could be seen. The samples were immediately placed in a jar with 10% buffered formalin. The piezotome was used to gently debride the walls of the cyst. The surgical site was flushed with copious amounts of 0.9% sterile saline and suctioned. The site was closed in two layers of 4-0 poliglecaprone in a simple interrupted pattern.

**Figure 3:**
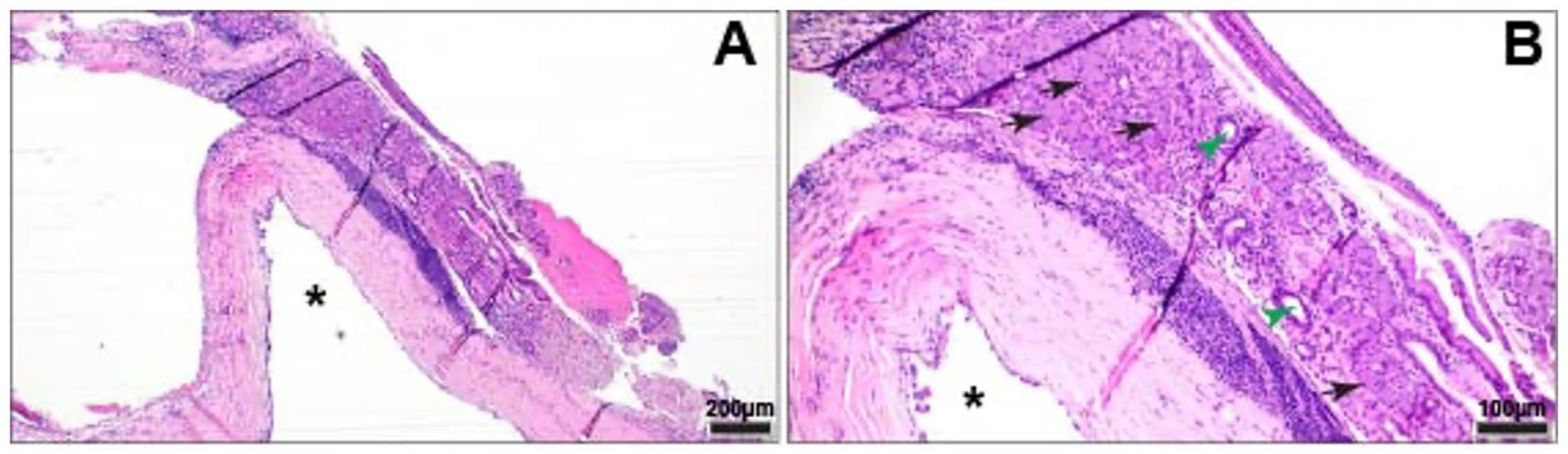
Cystic structure underlying acini (black arrows) and ducts (green arrow heads) of normal serous salivary gland tissue. **(A)** Cystic structure (*) lined by attenuated, simple columnar epithelium. HE. **(B)** Cystic structure (*) with underlying band of lymphoplasmacytic inflammation. HE.

The sample was submitted for histopathology, which showed normal, slightly compressed serous salivary gland tissue adjacent to a dilated epithelium-lined structure (cyst-like). This epithelial structure was thought to represent dilated ductal tissue (Figure 3). There was subepithelial lymphocytic and plasmacytic inflammation, with evidence of resorption and modeling in the adjacent orbital bone secondary to pressure and inflammation.

**Figure 4:**
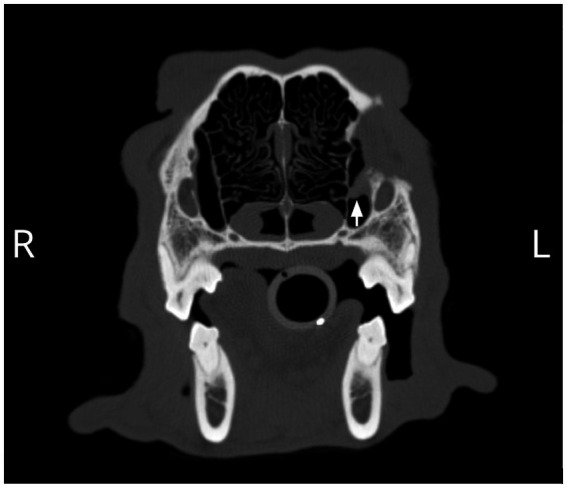
A remnant of abnormal tissue in the region of the nasolacrimal canal (arrow) and associated smooth regional osseous deformation resulting in asymmetry of the rostromedial wall of the left orbit was observed on the 4-month postoperative CT scan.

The patient returned 4 months later for a recheck CT. The owner reported bilateral mild, mucoid ocular discharge at home. There was adequate, symmetrical retropulsion of both eyes, and the left eye was in the correct position. Bloodwork showed a persistently elevated Alkaline phosphatase (ALP), which was similar to prior. The CT showed a small, tubular, fluid-attenuating, peripherally contrast-enhancing structure located at the region of the previously noted heterotopic salivary tissue (Figure 4). The cystic structure was 3.8% of the original volume (1.4 cm L × 0.5 cm W × 1.1 cm H; prior 2.5 cm L × 3.0 cm W × 2.7 cm H), indicating that a significant amount of the tissue was removed but that some heterotopic salivary tissue remained. The bony changes and local soft tissue swelling were static to mildly improved. No further specific surgical therapy was pursued.

## Discussion

This case report presents a salivary choristoma localized within the bones comprising the ventral orbit, representing the first documented report on imaging and management of this condition in this anatomical location in a canine patient. Salivary choristomas, also referred to as heterotopic salivary gland tissue or ectopic salivary tissue, are well documented in humans; however, the literature describing these in other species is lacking (3, 6, 7). Limited references exist regarding equine heterotopic salivary tissue (8, 9). One histopathological report of retrobulbar heterotopic salivary gland tissue in the dog has been previously presented (10).

Differential diagnoses for an intraorbital cystic lesion in the dog include a sialocele, dermoid cyst, lacrimal gland neoplasia, and inclusion cyst (11–14). In this case, a multimodal diagnostic approach incorporating advanced imaging, cytology, and histopathology was essential in distinguishing the lesion as a salivary choristoma. A critical aspect of diagnosis was determining the precise anatomic location of the lesion, potential association with adjacent structures, and cellular composition of the mass. The absence of contrast communication with nearby structures and the histologic characteristics supported the diagnosis of a salivary choristoma.

Differentiation between salivary choristomas and other cystic or glandular lesions is crucial, as treatment approaches vary. Treatment of heterotopic salivary tissue relies on surgical excision of the mass (8). In contrast, sialoceles—cystic outpouchings of normal salivary tissue—necessitate removal of both the lesion and the associated major salivary gland to prevent recurrence (15). Thus, accurate preoperative identification of the lesion is imperative to guide appropriate surgical intervention. Furthermore, complete excision is recommended due to the potential for aberrant salivary tissue to undergo neoplastic transformation and to preserve adjacent structures affected by the growth of the mass (8).

Histopathologic evaluation is instrumental in the diagnosis of heterotopic salivary tissue. Similar to traditional salivary tissue, salivary choristomas have glandular structures and columnar epithelial cells, but they lack the tubular drainage structures found in functional salivary tissue. Additionally, the present case illustrates the regional effects of salivary choristomas on nearby soft tissues and bone, providing additional support for the surgical removal of salivary choristomas.

Comparative literature of human cases show that heterotopic salivary gland tissue can be found in multiple locations throughout the body, including the orbit, nasopharynx, and middle ear (2, 6, 7). Proposed etiologies for salivary choristomas include aberrant embryologic development; heterotopia, or abnormal persistence of normal tissues in an ectopic location; or heteroplasia, or abnormal differentiation of local tissues (2). Further research should investigate the prevalence of salivary choristomas in alternative locations, investigate their embryologic origins with anatomic specificity, and establish optimal diagnostics and treatment protocols for this rare condition.

## Data Availability

The raw data supporting the conclusions of this article will be made available by the authors, without undue reservation.
